# The Good, Bad, and Ugly of Online Recruitment of Parents for Health-Related Focus Groups: Lessons Learned

**DOI:** 10.2196/jmir.2829

**Published:** 2013-11-11

**Authors:** Susan Quach, Jennifer A Pereira, Margaret L Russell, Anne E Wormsbecker, Hilary Ramsay, Lois Crowe, Sherman D Quan, Jeff Kwong

**Affiliations:** ^1^Public Health OntarioToronto, ONCanada; ^2^University of CalgaryCalgary, ABCanada; ^3^Division of Paediatric MedicineHospital for Sick ChildrenToronto, ONCanada; ^4^Bruyere Research InstituteOttawa, ONCanada; ^5^Trillium Health PartnersMississauga, ONCanada; ^6^Institute for Clinical Evaluative SciencesToronto, ONCanada; ^7^Dalla Lana School of Public HealthUniversity of TorontoToronto, ONCanada; ^8^Department of Family and Community MedicineUniversity of TorontoToronto, ONCanada; ^9^University Health NetworkToronto, ONCanada

**Keywords:** parents, data collection, communication, social media, Internet

## Abstract

**Background:**

We describe our experiences with identifying and recruiting Ontario parents through the Internet, primarily, as well as other modes, for participation in focus groups about adding the influenza vaccine to school-based immunization programs.

**Objective:**

Our objectives were to assess participation rates with and without incentives and software restrictions. We also plan to examine study response patterns of unique and multiple submissions and assess efficiency of each online advertising mode.

**Methods:**

We used social media, deal forum websites, online classified ads, conventional mass media, and email lists to invite parents of school-aged children from Ontario, Canada to complete an online questionnaire to determine eligibility for focus groups. We compared responses and paradata when an incentive was provided and there were no software restrictions to the questionnaire (Period 1) to a period when only a single submission per Internet protocol (IP) address (ie, software restrictions invoked) was permitted and no incentive was provided (Period 2). We also compared the median time to complete a questionnaire, response patterns, and percentage of missing data between questionnaires classified as multiple submissions from the same Internet protocol (IP) address or email versus unique submissions. Efficiency was calculated as the total number of hours study personnel devoted to an advertising mode divided by the resultant number of unique eligible completed questionnaires .

**Results:**

Of 1346 submitted questionnaires, 223 (16.6%) were incomplete and 34 (2.52%) did not meet the initial eligibility criteria. Of the remaining 1089 questionnaires, 246 (22.6%) were not from Ontario based on IP address and postal code, and 469 (43.1%) were submitted from the same IP address or email address (multiple submissions). In Period 2 vs Period 1, a larger proportion of questionnaires were submitted from Ontario (92.8%, 141/152 vs 75.1%, 702/937, *P*<.001), and a smaller proportion of same IP addresses (7.9%, 12/152 vs 47.1%, 441/937, *P*<.001) were received. Compared to those who made unique submissions, those who made multiple submissions spent less time per questionnaire (166 vs 215 seconds, *P*<.001), and had a higher percentage of missing data among their responses (15.0% vs 7.6%, *P*=.004). Advertisements posted on RedFlagDeals were the most efficient for recruitment (0.03 hours of staff time per questionnaire), whereas those placed on Twitter were the least efficient (3.64 hours of staff time per questionnaire).

**Conclusions:**

Using multiple online advertising strategies was effective for recruiting a large sample of participants in a relatively short period time with minimal resources. However, risks such as multiple submissions and potentially fraudulent information need to be considered. In our study, these problems were associated with providing an incentive for responding, and could have been partially avoided by activating restrictive software features for online questionnaires.

## Introduction

Study recruitment is a challenging aspect of health research. Strategic communication, planning, and marketing are essential for reaching potential participants. By identifying a target population’s demographic characteristics and interests, researchers can select methods that represent the best investment for study recruitment given the study objectives and budgetary, human resource, and time constraints [1,2].

Historically, researchers have relied on traditional methods, such as random-digit dialing, school or workplace recruitment, mass postal mailing, poster displays, and mass media campaigns, to recruit participants for research studies. However, these methods are often resource intensive and costly, sometimes with limited reach [3]. More recently, researchers have used the Internet to recruit younger participants for studies, by using paid advertising (eg, Google AdWords, Facebook Ads), links on websites, email lists, and online classified advertisement sites [4-6]. Compared with traditional strategies, Internet-based methods may achieve desired sample sizes with lower costs for distribution of materials [7]. They allow for rapid, convenient participation for study participants, and provide an easier means to share information across social networks, as well as an efficient process to respond to inquiries. Internet-based recruitment methods can be used to target difficult-to-reach groups who cannot be accessed by traditional means (eg, highly mobile individuals, those without a landline telephone) provided those groups have Internet access [8]. Further, respondent anonymity may decrease bias regarding sensitive health issues [5,9,10]. In contrast, anonymity can also reduce an individual’s fear of behavioral consequences, resulting in decreased honesty and increased inappropriate activity from the perspective of the researcher, such as creating multiple identities and/or falsifying information for personal benefit [11]. This type of activity, termed “gaming the system,” may be related to offering an incentive [12].

In 2012, we began recruitment for a qualitative study to examine and understand parental perceptions of the advantages and disadvantages of expanding influenza immunization programs to include delivery to children in elementary schools in Ontario, Canada, as well as the programmatic characteristics that would facilitate its adoption in this province. Given that eligible participants were parents of school-aged children, and likely busy individuals within the age range from early 20s to middle age, we used multiple advertising modes and conventional mass media to identify and recruit participants for focus groups.

In this paper, we evaluate the impact of the recruitment methods on study recruitment response, and compare the relative efficiencies of different advertising modes. The aims of this paper were to (1) compare the demographic attributes of eligible participants who completed the questionnaire during an incentive versus a nonincentive recruitment period; (2) compare questionnaire response patterns between multiple and unique submissions; (3) assess the uptake, defined as the number of times advertisements were viewed among eligible participants who made unique submissions; and (4) measure efficiency, defined as the average time invested by study personnel to recruit a unique eligible participant by advertising mode.

## Methods

### Participants

This is a descriptive study of data collected from an online questionnaire used to screen participants for a qualitative study on parental perceptions of school-based influenza immunization. Participants satisfied eligibility criteria if they were (1) English language proficient; (2) resided in Ontario, Canada; (3) parents or guardians of at least 1 child between the ages of 4 and 18 years in an Ontario school; and (4) involved in decisions about their child’s immunizations.

### Procedures

We invited participants to complete a 5-minute online questionnaire that had been pretested for usability and technical functionality. From October 9 to 11, 2012 (period 1), we offered a CAD $5 Amazon electronic gift card as an incentive to those participants who met the self-reported eligibility criteria and completed the questionnaire. Because of the unexpectedly high volume of submissions received, we temporarily closed the questionnaire on October 11, 2012. The questionnaire was reopened from November 16, 2012 to February 17, 2013 (period 2) to recruit additional participants. In mid-January 2013, we focused on recruiting participants from rural areas. During period 2, no incentive was provided and software features were activated to permit submission of only 1 questionnaire per Internet protocol (IP) address.

Ethics approval was obtained from the University of Toronto Health Sciences Research Ethics Board and the Bruyère Continuing Care Research Ethics Board.

### Online Questionnaire

When participants entered the study website, they were asked to review information about the study. Participants were directed to an initial 5-question eligibility screen that asked whether they read English, wrote in English, resided in Ontario, were parents or guardians of at least 1 child between the ages of 4 and 18 years in an Ontario school, and were involved in decisions about their child’s immunizations. Another question asked participants how they heard about the study. The software automatically directed eligible individuals to the rest of the questionnaire, whereas those who were ineligible were directed to a page that prevented them from accessing the questionnaire. Participants could quit the questionnaire at any time. The full questionnaire included questions about number of children, the grade level of each child, whether participants or their children had previously received influenza immunization, and questions about participant’s age, sex, postal code, marital status, education, and ethnicity. Participants were asked if they were willing to engage in a focus group. If they agreed, they were prompted to indicate the type of focus group they preferred (teleconference or Internet forum) and to provide their contact information (name, email, and phone number). Only those who agreed to participate in the focus groups were required to provide their names. Email addresses were required when the incentive was offered or when participants agreed to engage in the Internet forum focus groups. Each page of the questionnaire had 1 to 3 questions, and there were a maximum of 17 pages for those who agreed to participate in the focus groups. FluidSurveys Version 4.0 (Ottawa, ON, Canada) was used for data collection and data were subsequently exported into Microsoft Excel 2010 and Stata SE 10.0 (StataCorp LP, College Station, TX, USA) for analysis.

### Recruitment

Our primary recruitment strategy involved several free online advertising modes. These were (1) social media (Twitter and Facebook), (2) online classified advertisement websites (Craigslist and Kijiji), (3) advertisements on deal forum websites (RedFlagDeals, SmartCanucks), and (4) postings to public health email lists and websites. As a result of these efforts, we were contacted by conventional mass media outlets who further publicized the study. In the last month of period 2, we also issued media releases to rural newspapers. Table 1 contains a list of the advertising modes and number of advertisements. Multimedia Appendix 1 includes a sample of the text used for the online advertisements.

**Table 1:** Advertising activities to recruit participants.

Advertising mode and source		Number of advertisements
**Social media**		
	Twitter	57
	Facebook	339
**Online classified advertisement websites**		
	Kijiji	71
	Craigslist	57
**Deal forum websites**		
	SmartCanucks	7
	RedFlagDeals	2
**Press releases to conventional mass media**		
	Rural newspapers	3 news articles in rural papers

### Online Advertising Modes

#### Social Media

We created and used a list of public health organizations, popular parent blogging groups or individuals (eg, @Canadian_moms, @ParentSource), parent magazines or websites (eg, Today’s Parent), local and national news agencies, physicians, and health reporters to follow and connect with on our social media channels.

We created a Facebook page (School Flu Shots-Ontario Study, created in September 2012) because Facebook is the most popular social media website in Canada [13]. We posted information about the study, such as the purpose, our research team, updates, and links to the study’s website and questionnaire [14]. Messages were posted between 1 pm and 4 pm on weekdays, the peak Facebook activity hours, on a weekly or biweekly basis during periods 1 and 2 [15].

Twitter is a social networking service that allows users to send and resend text messages within a 140-character limit. We created a Twitter account (@SchoolFluShots) and posted messages about the study and questionnaire links to generate interest. We tweeted twice per week during periods 1 and 2, and every weekday during the rural recruitment period (the last month of period 2). We also spent time monitoring conversations and identifying new groups and individuals with whom to connect.

#### Online Classified Advertisement Websites

Ads were posted on Craigslist and Kijiji, free classified advertisement websites that are used widely in Canada to advertise goods and services. We posted messages and links about the study in the “Jobs” and “Volunteers” categories for each of the 20 and 30 Ontario cities with listings pages on Craigslist and Kijiji, respectively. The ads were monitored daily over periods 1 and 2, and were reposted once they no longer appeared on the first page of results for the category, which was every 7 to 10 days for most cities.

#### Deal Forum Websites

SmartCanucks and RedFlagDeals are popular deal forum websites that advertise coupons, promotions, events, and freebies. Users can post messages on different threads that can be viewed by all website visitors. At the beginning of period 1, we posted 2 messages on RedFlagDeals under the “Freebies” and “Parenting” categories. We posted 28 messages on SmartCanucks under the “Paid Surveys and Mystery Shopping,” “Canadian Parents,” and 21 individual Ontario city categories. The ads in Smart Canucks were later merged into 7 ads and remained active during periods 1 and 2. On a daily basis, we monitored both RedFlagDeals and SmartCanucks for new replies from community members.

#### Public Health Email Lists and Websites

We sent emails to 148 Ontario health care organizations requesting they assist with study recruitment by sending a generic study email to their email list or posting a message on their own websites. We did not follow up to identify the number of organizations that complied with this request.

#### Press Releases to Conventional Mass Media

In the last month of period 2, we issued media releases to 35 rural newspapers, which resulted in 3 news articles (2 online and 1 paper) about the study.

#### Unsolicited Mass Media Contacts Resulting From Online Classified Advertisements

During period 2, we were contacted by the Canadian Broadcasting Corporation (CBC) requesting an interview. Our press releases had not targeted this news agency; the contacts were initiated as a result of our advertising on Kijiji. This resulted in 2 interviews that, in turn, led to 4 CBC news items (eg, online articles and videos, radio coverage, and television coverage) and 1 additional interview by a local Ottawa, Ontario, newspaper. The newspaper published an online video and a news article.

### Analysis

To examine the effects of the incentive and software restrictions, we compared paradata (ie, data regarding the survey process), such as survey completion time, IP address, country of the IP address, and missing data, and questionnaire responses for the period when we provided an incentive with no software restrictions (period 1) to the period without an incentive and software restrictions activated to limit access to the questionnaire (ie, single questionnaire per IP address) (period 2). We validated the self-reported Ontario residential status from the eligibility screen by examining the country of origin of the IP address (Canada vs elsewhere), and the self-reported postal code. FluidSurveys automatically checks an online database of IP addresses from different countries to determine the location of the IP address (email communication, June 2013). If the country for the IP address and the postal code were missing, we did a manual reverse lookup of IP addresses to determine if they were from Ontario by using an online IP address reverse lookup website [16]. We estimated the proportion of submissions originating from validated Ontario residents (ie, both postal code and IP address from Ontario).

We compared the distribution of questionnaires by advertising mode, median time for questionnaire completion, and the proportion of missing responses for demographic questions between multiple versus unique submissions. We classified the questionnaires as multiple submissions if more than 1 submission was received from the same IP address or if the email address was classified as nonunique. We used automated software procedures to check for multiple submissions from the same IP addresses and for nonunique email addresses. Email addresses were classified as nonunique if the same email address was (1) included on more than 1 submission even if originating from a different IP address, or (2) the email address was a close variant of another submission (eg, jim.doe@gmail.com and jim.doe@yahoo.com) based on a manual scan of each email address by 1 reviewer.

Bivariate analysis of statistical significance of time was performed using the Wilcoxon rank sum test. Proportions were compared using chi-square tests or Fisher exact tests. *P* values are presented for descriptive purposes. Stepwise logistic regression was used to identify demographic variables that differentiated unique eligible participants that were offered (period 1) or not offered the incentive (period 2). The independent demographic variables included age group (categorical), sex, marital status (single or married), self-identified ethnicity (categorical), and educational attainment (categorical). We also examined an adjusted model that included a variable for rural residence within the province of Ontario. *Rural* was defined as having a postal code with zero in the second position of the 6-digit postal code [17].

To evaluate uptake, we tracked the number of times advertisements posted on Kijiji, RedFlagDeals, and SmartCanucks were viewed. As indicators of social media popularity, we tracked the number of likes and friends on Facebook. We also tracked the number of retweets, mentions, and followers on Twitter. We could not track the number of views for the media-related activities, email lists, or websites. Respondents self-reported the source from which they heard about the study based on a close-ended checklist with the following categories: Facebook, Twitter, Kijiji, Craigslist, RedFlagDeals, SmartCanucks, email lists, word of mouth, friends or family, websites, and other. Those who checked other or website were asked to provide more details. From this, we calculated the proportion of completed questionnaires from unique eligible participants by advertising mode.

To assess the efficiency of each advertising mode, we summed the number of personnel hours required to advertise and monitor responses for each source, and divided this by the number of unique eligible participants identified using that source.

## Results

### Overview

**Figure 1:**
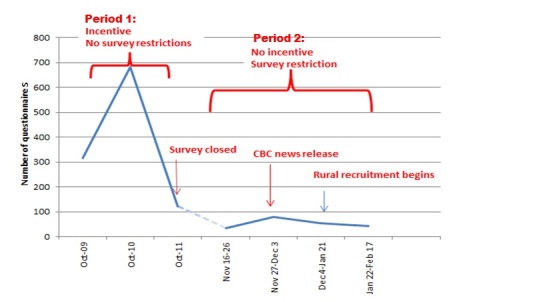
Study timeline showing questionnaire submissions in relation to the use of incentives and survey restrictions throughout the study (October 2012-February 2013).

During the 5-month recruitment period, we received 1346 questionnaires. During period 1 (incentive offered, software restrictions inactive) 1124/1346 (83.51%) were received. When recruitment reopened (period 2), 222/1346 (16.49%) were received. This increased minimally when several study-related media stories were published online (Figure 1).

**Figure 2:**
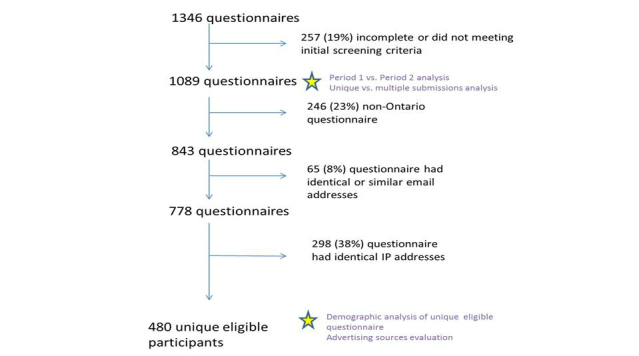
Participant flow diagram.

Of the 1346 questionnaires, we excluded 257 (19.09%) because they were incomplete (n=223) or because the potential participants did not meet the initial eligibility criteria (n=34), leaving 1089 for further analysis. We also excluded 246 that were not from Ontario based on the IP address and postal code, 65 based on similar or identical email addresses, and 298 based on nonunique IP addresses. This left 480 questionnaires from unique participants who were eligible for the qualitative study of school-based influenza immunization for which we had been recruiting participants (Figure 2). Of the 246 (22.6%) questionnaires that were not from Ontario, 26 (10.6%) were from other Canadian provinces, 182 (74.0%) were from the United States, and the remaining 38 (15.4%) were from outside North America (eg, India, Japan, United Kingdom, China).

Of the 1089 questionnaires retained for further analysis, 636 (58.40%) were from unique IP addresses and 453 (41.60%) originated from 118 nonunique IP addresses (ie, were classified as multiple submissions). Most of the nonunique IP addresses (105/118, 89.0%) occurred 2 to 5 times. Five nonunique IP addresses occurred 17 to 30 times. Of the 1089 questionnaires, 932 (85.58%) listed unique email addresses, 62 (5.69%) were missing email addresses, and 94 (8.63%) listed 39 nonunique email addresses. Most of the nonunique email addresses (32/39, 82%) occurred twice, whereas 7 occurred 3 times, and 1 email address occurred 5 times. Approximately one-third of the nonunique email addresses were variations of other email addresses and were only detected by manual review.

### Effects of Incentives and Software Restrictions (Period 1 vs Period 2)

Compared to period 1, period 2 was associated with a larger proportion of questionnaires being submitted from Ontario (92.8%, 141/152 vs 75.1%, 702/937, *P*<.001), and a smaller proportion of same IP address submissions (7.9%, 12/152 vs 47.1%, 441/937, *P*<.001). Of the 12 questionnaires submitted during period 2 that originated from nonunique IP addresses, 10 (7%) were from IP addresses from which submissions had also been received during period 1. Similar proportions of submissions from unique email addresses were received during both period 1 and period 2 (86%, 806/937 vs 87%, 132/152, *P*=.74). The median time for questionnaire completion was significantly longer for responses submitted during period 2 compared to period 1 (266 seconds vs 181 seconds, *P*<.001).

### Multiple vs Unique Submissions

The median time to complete a questionnaire for multiple submissions was less than that for questionnaires that were unique submissions (166 seconds vs 215 seconds, *P*<.001). We examined the time interval between submissions that were classified as multiple submissions (n=469) on the basis of IP address or email address: 272 (58.0%) were submitted within 5 minutes of the previous submission, 375 (80.0%) were submitted within an hour, and 450 (95.9%) were submitted within 24 hours.

Questionnaires from multiple submissions had a larger proportion of missing data than those from unique submissions (15% vs 8%, *P*=.004) (Table 2).

Among unique eligible participants, higher proportions of persons aged 40 years or older (39.2%, 49/125 vs 27.0%, 96/355), of female sex (83.2%, 104/125 vs 41.1%, 146/355), and of self-reported white ethnicity (70.4%, 88/125 vs 43.9%, 156/355) completed the questionnaire during period 2 compared to period 1 (Table 3).

In the adjusted model, sex and ethnicity were significant; females were more likely to participate during period 2 compared to period 1 (Table 4), whereas persons who self-identified as being of Chinese ethnicity had lower odds (OR 0.05, 95% CI 0.01-0.20) of participating during period 2 compared to those of white ethnicity. No other demographic variables were significant at the .05 level in the multivariate model. The adjusted model that also included rural versus urban residence within the province of Ontario showed similar results (Table 3). Participants living in urban locations had lower odds of participating during period 2, which was consistent with the study’s recruitment goal to target individuals from rural areas during the last month of the study.

### Uptake of Advertising Modes

During period 1, more than half of participants who completed questionnaires (192/355, 54.1%) were recruited through RedFlagDeals, whereas Craigslist or Kijiji, conventional mass media, and public health email lists or websites comprised the smallest contributions (<2% combined) (Table 5). However, during period 2, 28.0% (35/125) of participants who completed questionnaires were recruited through conventional mass media, 20.0% (25/125) through Craigslist or Kijiji, and 6.4% (8/125) through RedFlagDeals.

### Efficiency of Advertising Modes

Recruitment from RedFlagDeals was the most efficient, requiring 0.03 hours of staff time per questionnaire (Table 6). Two ads were posted on RedFlagDeals at the beginning of the study that resulted in 5077 views. In contrast, recruitment via Twitter was the least efficient, requiring 3.64 hours per questionnaire.

**Table 2:** Proportion of missing data for each demographic variable.

Variable	Multiple submissions, n (%) n=469	Unique submissions, n (%) n=620
Postal code	176 (37.5)	80 (12.9)
Education	48 (10.2)	25 (4.0)
Age	25 (5.3)	32 (5.2)
Single status	28 (6.0)	49 (7.9)
Ethnicity	75 (16.0)	49 (7.9)
Total	352 (15.0)	235 (7.6)

**Table 3:** Demographic characteristics of unique eligible participants^a^ (n=480).

Characteristic		Period 1, n (%) n=355	Period 2, n (%) n=125
Sex (female)		146 (41.1)	104 (83.2)
**Age (years)**			
	<20	2 (0.6)	1 (0.8)
	20-29	81 (22.8)	11 (8.8)
	30-39	153 (43.1)	60 (48.0)
	≥40	96 (27.0)	49 (39.2)
	No answer	23 (6.5)	4 (3.2)
**Marital status**			
	Single parent	50 (14.1)	19 (15.2)
	Married	262 (73.8)	106 (84.8)
	No answer	43 (12.1)	0 (0)
**Ethnicity**			
	White	156 (43.9)	88 (70.4)
	Chinese	84 (23.7)	3 (2.4)
	South Asian	36 (10.1)	8 (6.4)
	Southeast Asian/Filipino	14 (3.9)	2 (1.6)
	Korean/Japanese	6 (1.7)	1 (0.8)
	Mixed	7 (2.0)	7 (5.6)
	Other (Arab, Black, Latin American, West Asian, Aboriginal)	16 (4.5)	12 (9.6)
	No answer	36 (10.1)	4 (3.2)
**Education**			
	High school or less	29 (8.2)	6 (4.8)
	At least some postsecondary education (eg, college, university)	307 (86.5)	117(93.6)
	No answer or other	19 (5.3)	2 (1.6)
**Location**			
	Rural^b^	8 (2.3)	22 (17.6)
	Urban	301 (84.8)	101 (80.8)
	No answer	46 (13.0)	2 (1.6)

^a^Unique eligible participants exclude those identified as multiple submissions or those who did not meet the geographical criterion (Ontario residence).

^b^Rural location was based on the second digit of the 6-digit postal code being zero (eg, N0P 1L0) [17].

**Table 4:** Adjusted odds ratios^a^ comparing demographic characteristics of unique eligible participants^b^ from period 2 to period 1.

Characteristic		Adjusted OR (95% CI)	*P* value
Sex (female)		7.67 (4.12-14.27)	<.001
**Age (years)**			
	<29^c^	1.00	
	30-39	1.56 (0.67-3.56)	.29
	≥40	2.26 (0.96-5.29)	.06
**Marital status**			
	Married	1.00	
	Single	0.70 (0.34-1.41)	.32
**Ethnicity**			
	White	1.00	
	Chinese	0.05 (0.01-0.20)	<.001
	South Asian	0.65 (0.25-1.67)	.37
	Southeast Asian/Filipino	0.37 (0.07-1.92)	.24
	Korean/Japanese	0.46 (0.04-5.13)	.53
	Mixed	2.28 (0.65-7.96)	.20
	Other (Arab, Black, Latin American, West Asian, Aboriginal)	2.26 (0.96-5.29)	.06
**Education**			
	High school or less	1.00	
	At least some postsecondary education (eg, college, university)	2.22 (0.78-6.29)	0.133

^a^Adjusted odds ratio simultaneously adjusted for all variables listed in the table.

^b^Unique eligible participants excludes those identified as multiple submissions or those who did not meet the geographical criterion (Ontario residence) (n=390).

^c^<20 years was combined with 20-29 years because of the small sample size.

**Table 5:** How unique eligible participants heard about the study (n=480).

Advertising mode	Period 1, n (%) n=355	Period 2, n (%) n=125
RedFlagDeals	192 (54.1)	8 (6.4)
Friend or family	50 (14.1)	6 (4.8)
Facebook	47 (13.2)	21 (16.8)
SmartCanucks	17 (4.8)	10 (8.0)
Word of mouth	16 (4.5)	5 (4.0)
Twitter	11 (3.1)	2 (1.6)
Prefer not to answer	10 (2.8)	2 (1.6)
Public health email list or website	5 (1.4)	11 (8.8)
Craigslist/Kijiji	4 (1.1)	25 (20.0)
Press releases to conventional mass media	0 (0)	35 (28.0)

**Table 6:** Hours of staff time required for submissions from each advertising mode, the number of unique eligible participants^a^ recruited from each mode, and the efficiency (number of staff hours per questionnaire) and uptake of each mode.

Advertising mode and source		Hours, n	Unique eligible participants, n	Efficiency	Uptake
**Social media**					
	Twitter	47	13	3.64	13 retweets, 15 mentions, 112 followers, 469 following
	Facebook	37	68	0.54	16 likes
**Online classified advertisement websites**					
	Kijiji/Craiglist^b^	22	29	0.77	1193 views (Kijiji only)
Conventional mass media		4	16	0.25	Unable to assess
Email lists or websites		3	13	0.23	Unable to assess
**Deal forum websites**					
	SmartCanucks	6	26	0.22	3579 views
	RedFlagDeals	6	202	0.03	5077 views

^a^Total number of unique eligible participants reported is less than the number of unique eligible participants(367/480) because some participants provided no response or provided a response that could not be linked with a specific source(eg, friends or family, word of mouth).

^b^Craigslist did not provide any information on the number of views.

## Discussion

### Principal Results

By using multiple online advertising strategies, we recruited a large sample of participants in a relatively short time period with minimal resources. Although the Internet was an effective recruitment medium, we also observed a significant amount of suspected gaming during the recruitment process because some participants submitted multiple questionnaires. In contrast to those who made unique submissions, people who made multiple submissions from the same IP address or email address spent less time per questionnaire and their responses had a greater percentage of missing data. Further analysis revealed that approximately one-quarter of those who completed the questionnaire were not from Ontario, despite passing the initial eligibility screen of self-reported Ontario residence. Females were more likely to participate in period 2 (no incentive) compared to period 1 (with incentive), whereas persons who self-identified as Chinese ethnicity were less likely to participate in period 2 compared to those of self-identified white ethnicity. Recruiting participants via RedFlagDeals was the most efficient in terms of research staff time and had the highest uptake, whereas Twitter was the least efficient. The anonymity feature of online questionnaires may allow individuals the freedom to engage in behavior undesirable to researchers, such as completing questionnaires multiple times or providing false information. These behaviors are probably based on a desire to receive the maximum amount of an incentive possible, rather than a primary desire to contribute to the actual research. Failure to identify this type of behavior can compromise data integrity. Many of our findings and recommendations (summarized in Textbox 1) are consistent with those included in the Checklist for Reporting Results of Internet E-Surveys (CHERRIES) statement as ways to prevent and detect multiple entries from the same individual [18]. We have identified several additional methods to help ensure authentic data are obtained from unique persons beyond enabling cookies and conducting IP checks.

**Recommendations for researchers and designers for Internet-based questionnaires.**

- Include a disclaimer stating that any activity such as completing the questionnaire multiple times will be considered to be suspicious, the entries classified as invalid, and that no incentive will then be provided.
- Activate restrictions such as cookies or IP address restrictions to prevent participants from submitting multiple questionnaires.
- Include logic checks and repeated questions with definite answers (eg, username, birth date) throughout the questionnaire.
- Choose an incentive that is accessible and appealing to the geographical target. If there are sufficient funds in the budget, use post office mail instead of email to deliver the incentive. This provides another opportunity to validate a geographical criterion and detect if multiple submissions are present.
- Examine the paradata (eg, questionnaire completion time, IP address) to identify inappropriate activity or gaming.
- Deploy questionnaire-specific links for each advertising mode (multiple site entry linkage) to help identify the questionnaire participant’s source, rather than relying on self-report.

### Gaming

Preventing multiple submissions is a significant challenge with online recruitment. Users can easily create multiple email accounts or use different computers (thus different IP addresses) to complete questionnaires several times to maximize receipt of an incentive. In this study, approximately 43.07% (469/1089) of our participants made multiple submissions, which is significantly higher than reported in other studies. In an online questionnaire of men who have sex with men (MSM), Bowen et al [12] detected that 33% of their sample made multiple submissions, with the largest contributions coming from participants classified as “infrequent” (2-5 submissions; 36% of all multiple submissions) and “hackers” (>30 submissions; 34% of all multiple submissions). In another study of Latino MSM, 10% of the respondents made multiple submissions and 55% of these came from 1 person [10]. Differences in these rates across studies may be explained by how multiple submissions were defined and identified, where the study was advertised, the type of survey restrictions implemented to control or limit multiple submissions, the value of the incentive, and the type of participants targeted. For example, to gain more exposure, we posted on the deal forum websites under the “freebie” and “paid survey” categories because these were popular. In retrospect, we suspect that posting under these categories may particularly attract people who are highly motivated to obtain incentives and, thus, are more likely to make multiple submissions. We found that incentives seemed to have the least impact on the response rate of women and the greatest impact on the response rate by individuals who self-reported as being of Chinese ethnicity. This may be of value to researchers who are recruiting individuals of a specific gender, race, or ethnicity, and would be worthwhile to explore further.

Providing an incentive has been associated with increasing the frequency of multiple submissions. Bowen et al [12] found that men who were designated as eligible for compensation were 6 times more likely to have multiple submissions than the unpaid group [12]. Similarly, we found that the proportion of questionnaires submitted from nonunique IP addresses during period 1 (incentive offered) was 6 times larger than during period 2, the no-incentive period (47% vs 8%, respectively). Although we also activated software restrictions during period 2 to prevent multiple submissions from nonunique IP addresses, we suspect the incentive was the main driver of multiple submissions because the proportion of questionnaires received from non-Ontario residents was significantly higher when there was an incentive and the use of software restrictions should not have impacted that. Participants who made multiple submissions from the same IP address or email address spent less time per questionnaire, generally completed all the questionnaires consecutively within a short period of time, and had a higher percentage of missing data in their responses. This is not surprising because participants who have completed the questionnaire multiple times are likely to be familiar with the questions and therefore take less time to complete subsequent submissions. Also, by consecutively filling out the questionnaires and skipping questions, participants can maximize the total value of incentives received in a relatively short period of time. This type of behavior can have detrimental effects on the research budget; we estimate that a total of CAD $1485 (297 CAD $5 giftcards) was spent on individuals who were likely gaming the system.

Several strategies have been recommended to prevent or minimize the occurrence of multiple submissions: requiring participants to provide a unique identifier at the start of the questionnaire; using questions with definite answers (eg, birth date, name, phone numbers) repeated in the questionnaire, including questions specifically designed to catch dishonest participants; activating software restrictions to prevent participants from entering the questionnaire from the same IP address; and enabling cookies to prevent participants from entering the questionnaire from the same computer station [7,19-21]. Although none of these strategies are perfect, they provide extra barriers to detect and prevent multiple submissions. Prior studies using social media and other online resources as recruitment tools have advised implementing a protocol which includes checking IP and email addresses and following up with telephone confirmations as needed to guard against this behavior [22,23]. Conducting a reverse lookup of IP address location or postal code (as we did) can also identify participants who are outside the geographical target.

### Incentive Type

We suspect that the amount and type of incentive in this study may have contributed to the high rate of fraudulent data (ie, submissions from outside Ontario although the respondent indicated Ontario residence) in our sample. The value of the incentive (CAD $5 gift card) may have been too high for simply completing a 5-minute questionnaire. We used electronic Amazon gift cards that can be used in a wide variety of jurisdictions because they were easy to distribute by email, which can be used to purchase a wide variety of items. It is possible that a small percentage of parents truly resident in Ontario might have been visiting outside of Canada when the questionnaire was completed, but we doubt that this was the case for most persons classified as not being Ontario residents. For studies that are attempting to recruit participants from a specific geographic area, we recommend the use of an incentive that is only locally accessible (eg, local grocery chain gift card). Alternately, incentives could be sent to recipients by first-class mail only because the post office will return them if the mailing address is not valid, providing another opportunity to validate geographic residence.

### Advertising Mode Evaluation

In this study, we found that the deal forum websites (RedFlagDeals and SmartCanucks) had the largest uptake while requiring the least amount of time to maintain or update. Online classified advertisement websites (Craigslist and Kijiji) were also helpful with recruiting participants, especially during period 2. A reporter from the CBC national news corporation contacted us after seeing the advertisement in Kijiji, which resulted in our first media exposure and generated subsequent interviews and media stories. Without this exposure, we might have had more difficulty recruiting during period 2, given there was no incentive and most participants only heard about the study through conventional media during this period.

Using social media (Facebook and Twitter) was not as efficient for study recruitment as we had anticipated. Previous studies have examined using Facebook for recruitment, but these studies relied on paid advertisements [5,6]. In our case, we disseminated recruitment information by social networks, of which most groups were professional health care organizations. We likely would have had better success with these recruitment methods if we were more widely known before our online presence or had a larger network of followers on social media. Given the novelty of using social media for study recruitment and the limited resources available, we were hesitant to engage with our audience on topics unrelated to study recruitment, which may have affected the interest level. Further, most of our communication was one-way and focused entirely on study recruitment, which may not have been appealing to our audience. Instead, it would have been helpful to actively engage with our audience about related topics of interest (eg, immunization, cold and flu tips). When developing a social media strategy for study recruitment, researchers need to consider how to balance the needs and interests of their social media audience with the study objectives.

### Strengths and Limitations

We explored a range of relatively novel advertising approaches for the purpose of recruiting parents for research. We also conducted automated and manual reviews to scan email addresses to identify multiple submissions. Although it was more resource intensive, manual methods allowed us to identify additional email addresses leading to identification of multiple submissions that would not have been detected by automated methods alone. To validate one of the eligibility criteria, we reviewed all the postal codes to ensure they were from Ontario and did a reverse lookup of IP addresses to verify an Ontario address when the postal code and country of IP address was absent. The limitation of relying on an IP address is that it can easily be manipulated or hidden. Ultimately, we successfully recruited all participants (n=55) needed for the focus groups with acceptable cost. Even so, a participant who self-reported being a parent during screening revealed that this was not true while participating in a focus group. This person left the focus group claiming she had not clearly understood this criterion for eligibility. This highlights the challenges in ensuring the validity of self-reported information, particularly for online responses.

We may have eliminated some legitimate respondents by removing questionnaires deemed to have been multiple submissions; it is possible that different people can share the same IP address. We may also have eliminated legitimate Ontario residents by removing questionnaires from non-Ontario IP addresses; some Ontario residents may have been traveling when responding or, because of the use of Web proxies, virtual private networks, and mobile networks, the IP address may not have been representative of the participant’s physical location. However, given that our analysis suggested gaming, we elected to err on the side of caution in retaining participants for focus group selection. The recruitment methods used in this study tended to attract a younger and more educated population, and should be used with caution if the main objective is to obtain a representative sample of parents. We defined a rural participant as someone who had a postal code with a zero in the second position of the 6-digit postal code. This indicates residence in an area that is not accessible by letter carriers. This may differ from definitions used outside of Canada; therefore, recommendations from rural participants in this study may be not be generalizable elsewhere.

A key limitation of online recruitment methods includes the inability to reach socioeconomically or educationally disadvantaged groups who may lack the skills to use, or have adequate access to, the Internet [24]. Further, although we asked participants how they heard about the study, the validity of this response may also be an issue because this was based on self-report. Such self-reports can be validated if the study design uses unique study URLs for each advertising mode (multiple site entry technique) [21]. We did not track the number of advertisements that public health posted about the study; therefore, we cannot fully assess the contribution from this mode. Additionally, because this stage of our study involved only a single brief questionnaire, we were not able to evaluate the effect of the advertising modes on retention rates. Such data would be very valuable to researchers. Finally, we conducted a series of post hoc analyses to examine differences between the patterns of response for different scenarios (unique vs multiple submissions, period 1 vs period 2), increasing the risk of type 1 error.

### Conclusions

Our study has identified that the Internet can be a useful way to recruit parents for research in a relatively short time with limited resources, but it also identified important elements that researchers need to consider in order to fully utilize the Internet for study recruitment. When studies are conducted face-to-face, participants may assume more accountability for their behavior than with research conducted online. The anonymity of online research could lead some individuals to engage in dishonest behavior from which they would otherwise refrain if they knew they could be easily identified. When incentives are linked to email addresses, it is easy for individuals to create new accounts to collect more than 1 incentive.

It is imperative for researchers to implement control measures in the study design and questionnaire programming stages to limit and detect gaming. As the Internet evolves and more individuals are accessible online, it will be critical for researchers and questionnaire designers to understand how best to address these challenges to ensure and preserve data integrity.

## Multimedia Appendix 1

Sample of text used for advertisements.

## Abbreviations

CBC: Canadian Broadcasting Corporation
CHERRIES: Checklist for Reporting Results of Internet E-Surveys
IP: Internet protocol
MSM: men who have sex with men
