# Supplementary material for: The Good, Bad, and Ugly of Online Recruitment of Parents for Health-Related Focus Groups: Lessons Learned
Source: J Med Internet Res. 2013 Nov 11;15(11):e250. doi: 10.2196/jmir.2829 (PMC3841369; doi:10.2196/jmir.2829)
Supplement: Supplementary file 1 [file jmir_v15i11e250_app1.pdf]

RedFlagDeals

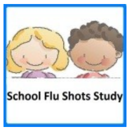

Subject: Free $5.00 Amazon.ca gift card for completing an online questionnaire(Ontario)

A team of researchers would like to find out what parents in Ontario think about the advantages and disadvantages of adding yearly influenza immunization (flu shots) to the immunizations that children get at school.

We want to hear about parents' experiences, opinions, or stories. Study results will help us develop recommendations to public health agencies, school boards and the provincial government.

Currently we are asking parents to complete a 10-minute online screening questionnaireto determine their eligibility to participate in a group discussion (focus group) that will take place at a later date.

We would like to talk with Ontario parents who:

- have at least one child currently enrolled in elementary, junior high, or high school;
- are most or jointly responsible for making health decisions for the children; and
- are able to speak or write in English

If you are eligible and complete the screening questionnaire, you will receive a $5 Amazon.ca gift card.

To learn more about the study and to access the questionnaire, click here: www.schoolflushots.ca

This study has been approved by the University of Toronto's Health Sciences Research Ethics Board and the Bruyère Continuing Care Research Ethics Board (Ottawa). This study is sponsored by Public Health Ontario and the Public Health Agency of Canada/Canadian Institutes of Health Research Influenza Research Network.
